# Opportunities to produce food from substantially less land

**DOI:** 10.1186/s12915-024-01936-8

**Published:** 2024-06-24

**Authors:** H. Charles J. Godfray, Joseph Poore, Hannah Ritchie

**Affiliations:** 1 Oxford Martin School, Oxford University, 34 Broad St, Oxford, OX1 3BD UK; 2 Department of Biology, Oxford University, 11a Mansfield Rd, Oxford, OX1 3SZ UK; 3 Our World in Data, Oxford University, 34 Broad St, Oxford, OX1 3BD UK

**Keywords:** Food system, Vertical farming, Meat substitutes, Cellular agriculture, Fermentation, Food processing

## Abstract

The vast majority of the food we eat comes from land-based agriculture, but recent technological advances in agriculture and food technology offer the prospect of producing food using substantially less or even virtually no land. For example, indoor vertical farming can achieve very high yields of certain crops with a very small area footprint, and some foods can be synthesized from inorganic precursors in industrial facilities. Animal-based foods require substantial land per unit of protein or per calorie and switching to alternatives could reduce demand for some types of agricultural land. Plant-based meat substitutes and those produced through fermentation are widely available and becoming more sophisticated while in the future cellular agricultural may become technically and economical viable at scale. We review the state of play of these potentially disruptive technologies and explore how they may interact with other factors, both endogenous and exogenous to the food system, to affect future demand for land.

**Supplementary Information:**

The online version contains supplementary material available at 10.1186/s12915-024-01936-8.

## Producing food using less land

**Fig. 1:**
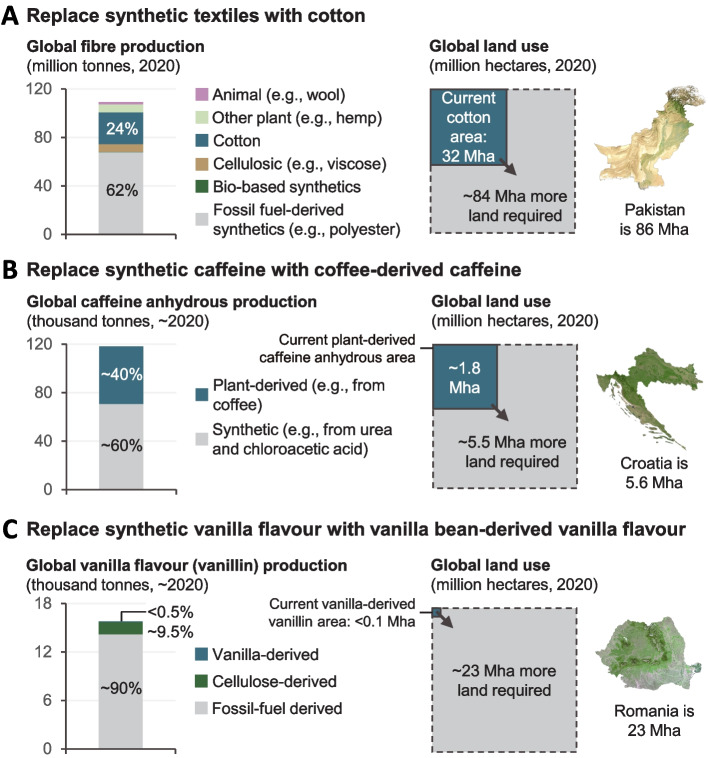
The land that would need to be brought into cultivation to compensate for existing near-landless technologies. The analysis is intended to be illustrative and assumes one-to-one substitution of natural for synthetic products (in reality the absence of a synthetic product would increase prices and reduce demand for the alternative)

Virtually, all the food we eat is produced on arable and livestock farms, and agriculture dominates land use in all but the coldest and driest parts of the world with major negative consequences for biodiversity, nutrient and pollutant runoff, and climate change [1]. But recent advances in biology and related sciences offer the prospect of producing some types of food using substantially less land than we do at present [2, 3]. This has happened before—demand for agricultural land would be substantially greater in the absence of a number of technologies—such as synthetic textiles and flavourings—that we have now (Fig. 1). How likely are these potentially disruptive new advances to translate into novel production systems that are commercially viable at scale? Were this to happen, what would be the consequences for the global food system and how we use land?

**Fig. 2:**
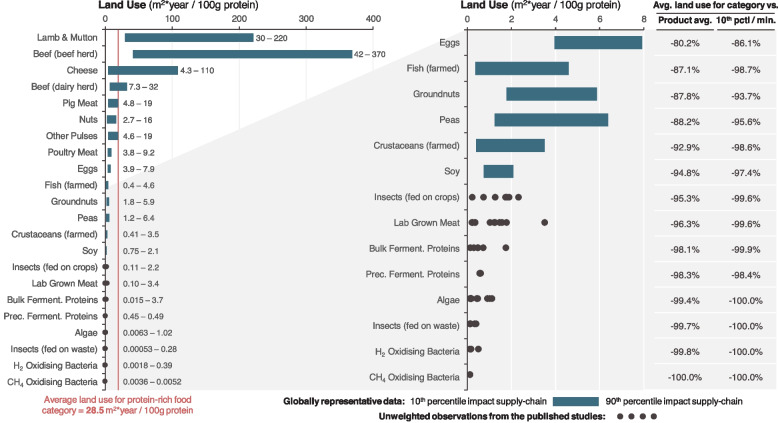
The land use of protein-rich foods. For crops, land use is calculated as the inverse of yield with a time correction for multi-cropping (more than one crop per year) and fallow duration (time in a rotation where land is left fallow). For animal products, land use includes grazed areas and feed. Urban land use (e.g. for solar energy) is also included where it is likely to represent over 10% of the total land use. The impacts of additional processing of novel proteins to make them into meat- or egg-replacing foods is excluded here, and land use may increase if this is included. Data and sources are provided in Additional file 1

Farming requires land because sunlight is the energy that powers most food production. We begin by exploring two food production systems that do not need the sun: vertical farming and the chemical synthesis of food or food precursors. Much protein we consume comes from farmed animals, but eating animal-sourced food is a less land-efficient way of utilising sunlight than eating plant-based food (Fig. 2). We discuss a series of technologies that might replace some animal-source food and reduce demand for pasture and cropland to grow animal feed: plant-based meat substitutes, fermentation, and cellular agriculture. These technologies often produce amorphous and unappetising products, so whether they are accepted at scale will depend on advances in food technology that we discuss next. We then investigate some issues around coffee, milk, and other liquids we consume. We finish by exploring how these technologies might be integrated within the global food system, the consequent change in demand for agricultural land, and the headwinds that may affect their development and deployment. Here, we concentrate on the land sparing effects of novel technologies, but note that continuing work on closing yield gaps and raising yield ceilings will also affect the demand for agricultural land. Also, our aim here is to explore the potential for using less land and we touch only briefly on the important economic and social welfare aspects of any such changes which rightfully will be important considerations for policymakers.

### Indoor and vertical farming

Small amounts of exotic fruit were produced during the Roman Empire in greenhouses employing mica and other natural glass-like minerals, but it was only in the seventeenth century when cheap plate glass became available that greenhouse production systems became widespread [4]. Since then, glass has been augmented by plastic, and in many modern systems, the indoor environment is tightly controlled to optimise plant growth independently of external conditions. Growth can also be enhanced using additional lighting or releasing pollinators, while some modern production facilities are positioned near sources of heat (power stations, for example) that also stimulate growth. Glasshouses deliver much higher yields per area than growing outdoors and can provide protection from pests, diseases, and weather extremes [5]. For example, greenhouse-grown tomatoes can have yields of over 500 tonnes per hectare, 15 or more times higher than outdoor-grown tomatoes [6].

A recent extension of greenhouse technology is vertical farming where crops are grown indoors on stacked horizontal or vertical surfaces [7]. Artificial light at wavelengths most appropriate for plant growth is provided, facilitated by recent advances in LED technology. Plants are grown in hydroponic or inert solid substates and are provided with their precise nutrient requirements that may change as they grow. The most sophisticated systems monitor multiple environmental and growth variables in real time and use digital-twin modelling techniques to optimise the plant’s environment. The diurnal and annual growth periods can be extended, with some species growing continuously, allowing more crops per year [8].

Vertical farming is used most widely for herbs, salad crops, and tomatoes, relatively high value, fast-growing crops with amenable growth forms [9]. Very high yields are possible, for example, for tomatoes over 1000 tonnes per hectare, more than 30 times what could be achieved outside. Experiments have shown that broadacre crops such as wheat can be grown in vertical farming systems with yields very substantially higher than when grown outdoors. Global average outdoor-grown wheat yields are 3 t ha^−1^, but an experimental vertical farm could produce 14 t ha^−1^ over a short growing season and 70 t ha^−1^ if the facility was run continuously all year [10]. Higher yields still might be obtained by optimising CO_2_ concentrations and light levels [11].

Vertical farming has substantial capital costs and is very demanding of energy which currently effectively restricts it to high-value crops [9, 12], and even here, the recent increase in energy prices after Russia’s invasion of Ukraine led to several start-ups failing [13]. Wheat grown in a vertical farm might be 50 times more expensive than outdoors. The high energy use also affects whether vertical farming produces fewer greenhouse-gas (GHG) emissions than outdoor farming. Studies of existing vertical farms found they performed better on many environmental outcomes but not on GHG emissions, in large part because they relied on non-renewable sources of energy [14, 15]. These calculations could change if the electricity supply became decarbonised, and if the crop land replaced by vertical farming was used for carbon sequestration, though land may also be required to produce renewable energy [16].

We conclude that for the foreseeable future indoor farming and related technologies will be confined to high value crops such as herbs and some vegetables. But as these account for just 4% of agricultural land area, it seems unlikely that this will lead to a substantial near-term reduction in demand for land.

### Avoiding photosynthesis

The need for plants to be grown in the open air, exposed to sunlight and ambient carbon dioxide, drives demand for agricultural land. But there are alternative synthetic pathways and different sources of carbon that can be used to produce major components of human diets. Some of these pathways are not new; during the Second World War when Germany had limited access to fats and oils, it synthesised margarine from coal derivatives [17].

An alternative to photosynthetic food production is to start with a simple carbon source such as ethylene or syngas (a mixture of hydrogen and carbon monoxide). The carbon it contains can be obtained from three major sources: CO_2_ in the atmosphere obtained by direct air capture (DAC), organic waste, or fossil fuels. Free CO_2_ has to be chemically reduced to become usable, an energy-expensive step [18]. Syngas and ethylene are already used as the basis to synthesis paraffins, fats, and lipids, and in principle all major macronutrient monomers could be derived from this source [19].

A major challenge to synthesising food chemically is the issue of chirality [20]. Many organic molecules exist as different mirror-image molecules (enantiomers) with nature privileging one form over its mirror image. Chemical synthesis typically produces a mixture of enantiomers which are expensive to separate. However, fats and oils are generally not chiral and are the most likely targets for production at scale, though costs of energy and associated GHG emissions remain a barrier [19]. If these could be overcome, then synthetic fat production could lead to significantly reduced demand for land. Oil crops are responsible for ~ 7% of agricultural land, and tropical crops such as palm oil are leading drivers of deforestation and are grown on land with great carbon sequestration and biodiversity restoration potential [21]. Synthetic carbon sources can also be used to produce feedstocks for microbial fermentation (see below) [22–25] which typically avoids issues associated with chirality and could replace a broad array of land-based food products.

We conclude there is the potential for non-photosynthetic fats and oils and fermentation feedstocks to impact demand for agricultural land, though further technological advances are likely needed to make it economically viable.

### Plant-based proteins

Meat production is a major source of GHG emissions and occupies large areas of land for grazing and to grow crops for animal feed [1, 6]. Meat consumption is constant or slightly declining in high-income countries, driven by environmental, health, and animal welfare concerns, but is increasing globally, particularly in middle-incoming countries [26]. Achieving net zero emissions will require dietary change, especially in the rich world, and the next few decades are likely to see increasing attention on reducing meat consumption [27–29].

There are already many plant-based meat substitutes on the market [30]. Lentils, peas, and other legumes provide alternative sources of proteins, while traditional products such as tofu and seitan (from wheat) are important protein sources in different cuisines. More recently, plant-based products that more faithfully replicate the taste and texture (“mouthfeel”) of processed meat have been developed [31]. Impossible Foods (founded 2011) produces a plant-based burger which mimics animal blood using leghaemoglobin found in legumes (produced using genetically engineered yeast) [32]. Beyond Meat (founded 2009) also market a plant-based burger with the blood effect produced using beet juice. The companies claim their products require less than 90% the land needed for traditional beef burgers [33]. These burgers are marketed at a relatively high price point, but cheaper plant-based meat substitutes are increasingly being used to substitute for processed meat in sausages, patties, and ready meals [31].

There are many potential plant-based sources of proteins that might be used as meat substitutes [34]. A barrier to their use is a poor understanding of molecular-structure–function relationships: how the amino acid sequence determines three-dimensional structure and hence its physicochemical properties as an ingredient and the resultant mouthfeel [35]. Recent advances in machine learning have greatly accelerated the determination of protein structure from sequence, and though a more challenging problem, related techniques are likely to revolutionise the study of interactions between proteins and between proteins and other compounds. Plant proteins do not have the same amino acid profile as animal proteins which can lead to dietary deficiencies [36]. Eating a diversity of proteins helps address this, and genetically manipulating the protein to make it more nutritious is a further option [37, 38].

The challenge to nations and companies of meeting their net zero pledges, and the availability of mature technologies, suggests a move to plant-based meat substitutes may lead to a substantial reduction for some types of agricultural land in the next few decades [39].

### Fermentation

Fermentation has been used for millennia to produce alcoholic beverages, bread, cheese, and products such as yoghurt, kimchi, and sauerkraut [40, 41]. Today, there is great interest and investment in different fermentation technologies that might produce food with a relatively small land footprint [42]. A broad range of technologies are being explored but they can loosely be divided into bulk or biomass fermentation and precision fermentation.

Bulk fermentation utilises fast-growing microorganisms in bioreactors to produce large quantities of required substances [43, 44]. The first applications were in industrial chemistry to produce compounds such as ethanol and organic acids, but in the second half of the twentieth century, protein for food began to be produced commercially. A leader was the British company Quorn whose technology, first marketed in 1985, was based on a fibrous mycoprotein derived from the fungus *Fusarium venenatum* whose properties facilitated its processing into meat substitutes [45]. In addition to other multicellular fungi, species of yeast, bacteria, and microalgae have all been studied as protein sources, though only a very small fraction of possible species have been investigated [42, 46].

Precision fermentation differs in that it uses microorganisms to produce desired proteins and other compounds [47–49]. Typically, a eukaryote gene is genetically engineered into a microorganism which is grown in bulk in a bioreactor, and then the desired compound is extracted. The enzyme chymosin (rennet) used in cheese-making is now largely produced from genetically engineered yeast, while pharmaceutical insulin comes from modified *E. coli* bacteria and yeast. The bloody look and mouthfeel of Impossible Food’s burgers is due to leghaemoglobin produced by genetically engineered yeast. A major challenge for cellular meat production is providing the right growth medium, and until recently this had required the use of foetal calf serum which is both expensive and to many has ethical challenges. Precision fermentation can be used to produce at least some of the essential growth-medium components required for cell and tissue culture.

The economics, sustainability, and scalability of fermentation depends on the feedstock used, as does their land footprint. Most commercially available products today use feedstocks that could be used directly for food or feed. Quorn, for example, uses carbohydrates derived from wheat and maize though the fungi is more efficient at producing protein than animals fed on similar feed [50]. The company ENOUGH is building a large *Fusarium* mycoprotein factory in the Netherlands beside a starch factory to use its side-stream products [51]. Other by-products such a molasses (from sugar production) and cellulose-rich biomass (from agriculture and forestry) can be used. In principle, microorganisms can use very recalcitrant feedstocks, but processing of cellulose-rich material is required to make it suitable for fast-growing species which can be costly and energy-demanding. “Waste-to-nutrition” involving many potential feedstocks could make a significant contribution to developing the circular economy [52].

Bacteria-based systems with a variety of gaseous feedstocks have also been investigated [41, 53]. Methanotrophic bacteria can produce protein from methane, which has been used to make aquafeed and animal feed though at the cusp of financial viability. Other bacteria if supplied with a mixture of hydrogen and oxygen and CO_2_ can fix carbon and produce protein. The hydrogen and oxygen are produced by electrolysing water which is energy intensity though might still result in lower emissions if surplus renewable energy is used. However, the explosive property of the gas mixture and its low solubility requires expensive bioreactor engineering.

There are significant opportunities for improving the efficiency of fermentation by developing better microbial strains (for example those that tolerate higher cell densities), improving bioreactor design and cultivation systems, and producing proteins more suitable for human and animal consumption [53]. This can be done directly by genetic engineering or other synthetic biology techniques or indirectly using artificial evolution to select for desired effects. There is also research on assembling the components of biochemical processes such as protein synthesis in cell-free bioreactor systems [54].

Though less advanced than plant-based substitutes, the production of meat substitutes and other products produced by industrial-scale fermentation is likely to grow and may lead to a significant reduction in demand for land over the next few decades.

### Food from cell and tissue culture

Winston Churchill predicted in 1931 that “Fifty years hence, we shall escape the absurdity of growing a whole chicken in order to eat the breast or wing by growing these parts separately under a suitable medium” [55]. This has not come to pass, but the last decade has seen massive investment in cultured meat with several companies becoming unicorns (attracting over $1B investment) [56].

Though there are many variants, most current proposals for cultured meat start with an animal biopsy to provide a potential cell line which may be further treated (“immortalised”) to prevent ageing [57]. For commercial production, the cells are allowed to grow and multiply in large bioreactors bathed in a suitable growth medium, using technology very similar to that employed in the production of monoclonal antibodies. The cells are then harvested as a slurry and further processed (see below) to produce protein-rich substitutes for processed meat products such as ground beef or chicken nuggets. Plant-based proteins, fats, flavourings, and additives may be added along the production chain. As of January 2024, a single (chicken) product of this type, marketed by Good Meat (a subsidiary of Eat Just), has obtained regulatory approval in Singapore and the United States [58].

**Fig. 3:**
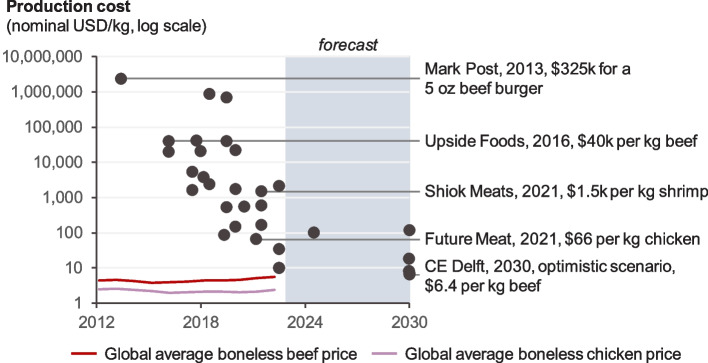
The production cost of lab-grown meat over time compared to global average chicken and beef prices. For lab-grown meat, data are from corporate press releases and from internal data from Systemic Capital (with permission). Data and sources are provided in Additional file 1. For chicken and beef, data are from FAOSTAT [67]

The nascent industry faces a variety of economic, engineering, and scientific challenges [59–64]. Existing growth mediums tend to be very expensive, and there are high capital costs to set up production facilities. Costs are coming down (Fig. 3) but from a high base and analogies to Moore’s Law for semiconductors may be too simplistic [63]. Growth media often include animal-derived components such as adult or foetal bovine serum that undermine animal-welfare arguments for cultured meat, though some progress has been made in finding non-animal replacements [65]. Mammalian cells grow more slowly than microorganisms and achieving high cell densities while avoiding contamination requires complex and expensive bioreactor design and control systems [66].

Products based on cell suspensions are a start but the hope of many researchers and companies in the field is to produce animal tissue or even whole muscles. Research on organ development and wound healing is providing many new insights into the underlying fundamental biology, but price-competitive commercial products are still some way off. A first step is to grow cells as sheets that can be harvested and stacked to give meat-like products. One company, Upside Foods (formerly called Memphis Meats) has regulatory approval in the US for a chicken product of this type (the second and only other product so far approved) though it is produced in very small quantities and not sold at a price reflecting its true costs [68]. A further step is to develop edible scaffolds on which cells can grow in a manner that mimics muscle tissue, possibly also allowing mechanical stretching as would occur in natural muscle development [69].

Cultured meat has a substantially smaller land footprint than real meat, even after considering the land required to grow its feedstocks (Fig. 2). Its other environmental footprints are difficult to gauge as no technology has yet been taken to scale, and emissions will depend critically on whether high energy inputs come from renewable sources [64, 70]. Whether regulators allow food-grade processing or demand more energy intensive pharmaceutical-grade processing (to remove possible toxins) will also be important [71].

We expect cultured meat to begin to replace some processed meat in the next decade, but the time scale for marketable textured meat (steaks etc.) is far less certain, and delivery will require known scientific barriers to be overcome. At least for the foreseeable future cultured meat is unlikely to have a major effect on demand for land.

### Food processing

Many novel ways of producing food that require less land give rise to relatively homogeneous substrates that need to be processed to provide the texture and mouthfeel we demand of food. A key process in current food technology is extrusion where a homogeneous ingredient mixture is forced through a die that shapes the product and introduces anisotropy (texture) [72]. In addition to the ingredient mix, temperature, pressure, and sheer stress can all be manipulated resulting in different outcomes. Current understanding of extrusion is in large part based on experiments as it is difficult to observe and then model what happens inside an extruder, though advances in semi-sold state modelling may allow a more predictive approach [34].

There are other ways to produce texture from a uniform mix including spinning and the use of shear cells [72, 73]. A particularly exciting technique is 3-D printing. As in other applications, 3-D printing involves robotically building a possible complex three-dimensional structure layer by layer [74, 75]. A paste or a powder may be deposited, typically followed by bonding using a binder or through heating. Most applications to date have involved niche areas such as sophisticated confectionary and patisserie creation and novel pasta shapes. But it also shows promise for potentially more widespread application including in meat substitutes. Fat and protein rich ingredients can be printed to mimic the distribution of muscle and fat in real meat. The relative ease with which printing parameters can be varied simplifies research into different structures and offers the prospect of tailoring meat substitutes to individual nutritional needs and preferences [34].

Though we have discussed a variety of different technologies separately in the preceding sections, combining them at the processing stage may allow the creation of more realistic alternatives. Several meat substitutes, for example, are hybrid products containing both plant-based and precision fermentation ingredients [76].

Advances in food processing do not directly affect land use but are important for facilitating the adoption and acceptability of novel food types which may compete with or replace land-based agricultural systems.

### Coffee, milk, and other liquid food and beverages

Coffee contains the stimulant caffeine but is a complex mixture of perhaps 1000 compounds, many of which contribute to the taste, aroma, and mouthfeel of the beverage [77]. The world consumes 2 billion cups of coffee a day with increasing demand contributing to tropical deforestation [78]. Chicory has been used for 200 years as a coffee substitute [79], but modern food science is allowing beverages to be created which much more closely resemble coffee. In principle, all components could be chemically synthesised, but the variety of start-up “bean-free coffee” companies are largely exploring different plant-derived substrates for roasting, including watermelon seeds, sunflower seed husks, date pits, lentils, and chicory—some by-products of existing production and some requiring new land [80]. Some companies use synthesised caffeine, but others use caffeine derived from tea—a crop that can also contribute to deforestation. At an earlier stage of development is coffee derived from cell cultures grown in bioreactors that produce bean tissue that can then be roasted [81].

Milk is a structurally more complex liquid, a colloidal dispersion of fat and oil droplets with globules of the protein casein in a liquid medium containing further proteins, lactose, vitamins, minerals, and other compounds [82]. The mouthfeel of milk is strongly influenced by its colloidal structure which is determined by the balance of the repulsive and attractive forces between droplets [34]. Plant-based milk substitutes, especially soy milk, have been produced for decades to meet the needs of vegans and those with lactose intolerance [83]. Their market share, diversity, and sophistication have increased markedly in the last 25 years, spurred by a greater shift to more plant-based diets and environmental concerns about farming animals [84]. But as important have been advances in food science that allow plant-based substitutes to have a very similar mouthfeel to real milk [85].

Milk is produced by cows and other ruminants which may be pasture or range fed, kept indoors and fed on plant-derived feeds, or a combination of the two. Because plant-based dairy substitutes require less land than animal-sourced foods, an increase in their consumption is likely to result in a net decline in demand for agricultural land. Exactly what types of agriculture land will be affected is harder to predict and will depend on whether pasture or grain-fed production is more displaced and the species of plant (soy, almond, oat, etc., for milk substitutes) used for substitute products that are most acceptable to consumers.

A blended egg is also a colloidal dispersion which when heated becomes a gel as the proteins it contains denature allowing the formation of hydrophobic and chemical bonds and hence particle aggregation [35, 86, 87]. Plant-based egg products seek to mimic this behaviour by selecting proteins that denature at similar temperatures and can form gels when combined with appropriate starches. However, most hens are fed on crop-derived feeds and are highly efficient converters of plant to animal biomass, so egg-substitutes are likely to have a relatively small effect on demand for land.

A mature market for non-dairy milk and creamers exists and is growing, and a smaller market exists for non-dairy cheese and eggs [88]. There are fewer coffee and tea substitutes, though this is an area of active research. Technological barriers for these types of new products seem lower than for other novel foods and were they to prove acceptable to consumers they could quite rapidly reduce the demand for some types of agricultural land.

### Land use and the food system

Our review shows that there are already technologies available to produce food with a reduced land footprint, and there is a high likelihood that further technologies will become available. How will these innovations interact with other food system factors to determine the global agricultural land footprint?

Human populations continue to rise though at a decelerating rate, and most demographers predict that numbers will peak this century and possibly begin to decline [89]. The major driver of this is the demographic transition where people are brought out of poverty, provided with reproductive health care and education for their children. Under these circumstances human fecundity naturally falls [90]. A reduction in poverty is obviously a good thing, but more wealthy people demand diets that require more resources to produce [91]. The peak demand for food from human population can be estimated in different ways, with most figures suggesting we will require to produce somewhere between 30 and 60% more food by mid-century than we do at present [92].

**Fig. 4:**
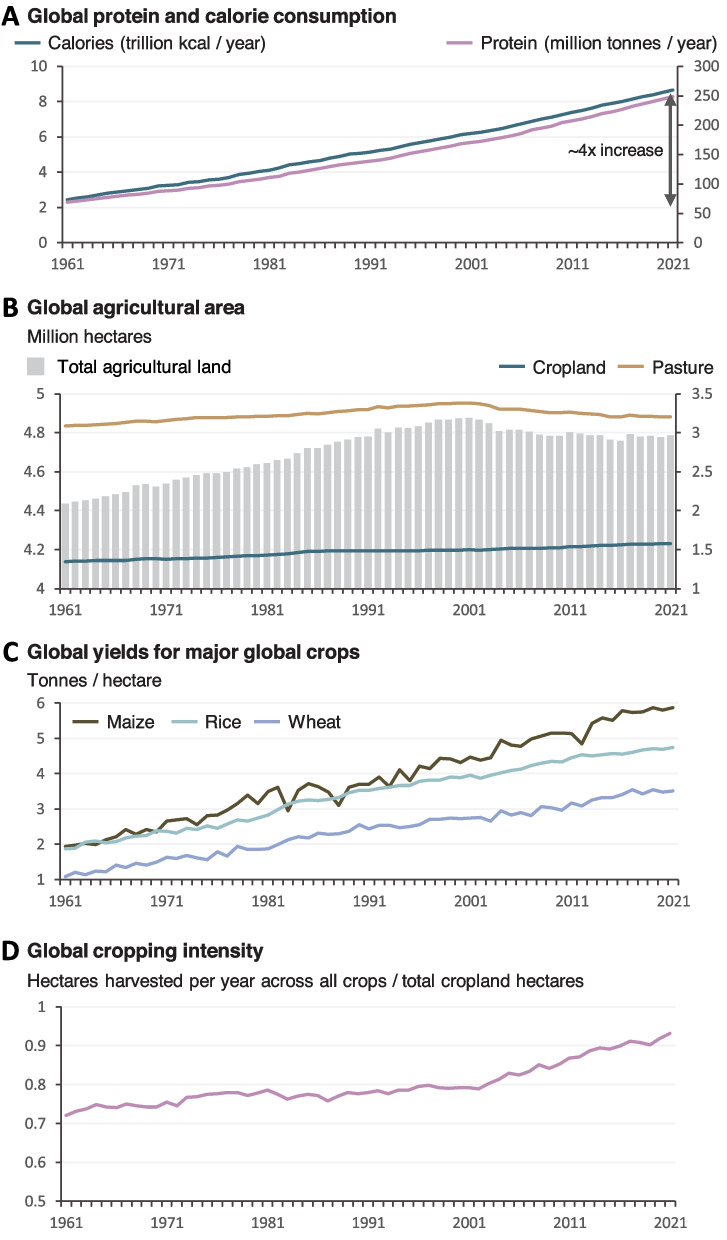
Trends in food production, agricultural land area, crop yields, and cropping intensity. Cropping intensity increases if the number of crops grown in a year increases (multi cropping) or the short fallow duration in a rotation decreases. Data from FAOSTAT [67]

Rising demand for food over the last 50 years has led to only modest increases in farmland thanks to increases in productivity (Fig. 4). Looking forward, we may be able to increase further the yield ceilings of major crops and especially more minor crops which have received much less research on genetic improvement. However, there are biophysical limits to crop productivity, and there is some evidence that these are being approached for some species [93]. Realised yields are often below those theoretically possible given local climates and soil. Closing this yield gap will be an important way of meeting future demand though a complex challenge requiring increasing farmer skills and often providing financial capital and access to markets [94, 95]. Considerable wastage occurs in the food chain, with estimates suggesting approximately a third of food is never consumed [96]. In low-income countries, losses in food supply chains are most important, while waste in the home, retail, and food service sectors is most important in high-income countries. Bearing down on waste, raising yield ceilings, and closing the yield gap will all lower the demand for food and for agricultural land. The degree to which we are successful in these goals will affect the pressures to develop landless food production systems.

Reductions in land available for agriculture will also spur research on alternative food production systems. Climate change may have some positive effects on agricultural production, but all integrated assessments suggest a net negative affect with some, possibly a large amount, of current agricultural land becoming unfarmable [97]. Growing populations and an increase in urbanisation will increase competition for land, and societies may wish to use some agricultural land for other purposes such as carbon sequestration and storage and providing habitats for biodiversity.

To summarise, a larger and wealthier global population will increase demand for food though the degree to which this is transmitted to increased demand for agricultural land will depend on progress in raising yield ceilings and closing the yield gap as well as reducing waste [94]. Climate change and competition for land will render some areas unsuitable for agriculture and increase the pressure to farm where possible. Newly wealthier people may demand more land-intensive food (particularly animal-sourced foods) though changes to diets with fewer GHG emissions may reduce pressure on land [98]. The net effects of these food-system processes affecting demand for land will determine investment in reduced and land-less production, though exogenous factors such as the cost of energy will also be influential.

### Headwinds

There are a variety of factors slowing the development of technologies that would reduce the demand for land. The most important is the economic challenges of producing food in new ways. Modern agriculture is very efficient because of generations of research, but also because some inputs are essentially free—sunlight for example—and because negative externalities are seldom internalised as part of the cost of food [99]. Thus, nitrogen run-off from agriculture causes extensive problems for drinking water and habitat quality, but the cost is typically born by society and not by the producer or consumer.

Conventional agriculture is also heavily subsidised and receives other indirect support. The World Bank has estimated that governments annually spend $0.75 trillion on agricultural subsidies [100]. At least in theory, this huge number provides enormous headroom to redesign global food systems in a way that have better health and environmental outcomes. However, it also implies a large number of vested interests that will resist change. S Vallone and EF Lambin [101] recently estimated that public financial support for animal production was 1200 and 800 times greater than that for novel technologies in the EU and US respectively. Lobbying by trade organisations and non-profits is also strongly skewed to support existing farming techniques.

One area of contention has been whether alternative foods can use the name of the product they seek to mimic in their labelling and marketing. Unilever, for example, in 2014 attempted to sue Eat Just (then trading under Hampton Creek) on the grounds that its product “Just Mayo” was misleading as it contained no eggs. In this case, however, the lawsuit was withdrawn, perhaps because a petition accusing the company of bullying attracted over 100,000 signatures [102]. Against this bias in favour of existing agriculture is the enthusiasm of venture capitalists for many landless-agriculture start-ups, some of which have been valued at many multiples of their plausible mid-term earnings.

There is understandable concern about novel technologies and calls to consume less animal-sourced products amongst people whose livelihoods depend on land-based agriculture. Many of these farmers are already relatively poor, and these fears have been pounced on by populist political movements, while food system transformation has become part of the “culture wars”. At the level of political economy, successfully meeting future food system challenges will require a just transition in which those with the least power are helped to adjust to inevitable change. An important facilitating narrative is of farmers as providers of both private goods (food sold in the market) but also public goods such as carbon sequestration and habitats for biodiversity that can attract state funding (which can be cost-neutral if subsidies are repurposed)—reduced land-based food production need not be associated with reduced small-farm income. It is also important to recognise that the incomes and nutrition of people in the poorest countries need to be raised to acceptable levels before they can be expected to contribute to reducing environmental threats, though there are opportunities for more sustainable technologies to be introduced earlier to avoid some of the problems of previous development pathways.

Public acceptance of novel foods will be affected by lobbying but also by the hard-to-predict dynamics of public opinion [103–108]. AE Sexton, T Garnett, and J Lorimer [109] used quantitative social-science methodologies to identify positive and negative narratives about alternative proteins. Different positive narratives stressed (i) health benefits, (ii) feeding the world more securely, (iii) reduced harm to the environment and animals, (iv) greater food safety and certainty of content, and (v) an enjoyable tasty food experience. Negative narratives highlighted (i) unnaturalness, (ii) that they are not a true narrative, and (iii) their irrelevance to feeding the world at scale. Interesting dissonances can be observed: individuals simultaneously privileging naturalness in foods but welcoming meat alternatives as reducing harm to animals and people arguing new technologies are at the same time irrelevant and a threat to jobs. These entwined narratives about alternative proteins and the other technologies discussed here will continue to interact and evolve and be subject to external forces such as changes in food prices and the cost of living as well as the degree to which a changing climate will incentivise individuals to alter their behaviour to reduce emissions.

## Conclusion

The imperative to make the global food system sustainable will almost certainly lead to radical change in the coming decades, and this is likely to include a move to reduced and land-less agriculture. Reaching net zero is very difficult without a switch to more plant-based diets, and this will reduce demand for land for pasture and feed, a move that will be accelerated as new technologies improve the acceptability of plant-based alternatives. A series of new technologies will also affect demand for land. Most mature is the production of protein-rich and other food components by microbial fermentation. Further in the future but a very active area of research is cellular meat. We believe products with both high greenhouse gas and biodiversity impacts—coffee, tea, cocoa, oil palm—will increasingly become targets for research on substitution. The energetic costs of land-less agriculture are high and at present make the substitution of staple food products unlikely, though this might change if cheap renewable energy resources became available. Were substantial amounts of land to be released from agriculture then some will certainly be used for urban expansion and other immediate human uses, but there would also be the excellent opportunity to repurpose it to mitigate climate change through carbon sequestration and to address the biodiversity crisis.

### Supplementary Information

Additional file 1: Fig. S1. a Replace synthetic textiles with cotton. b Replace synthetic caffeine with coffee-derived caffeine. c Replace synthetic vanilla flavour with vanilla bean-derived vanilla flavour. Fig. S2. Cultivated Meat Cost Curve. Meat Cost Curve. Fig. S3. Summary. Underlying studies.

## Data Availability

A review with details of data sources for figures given in the supplementary information.

## References

[1] Foley JA, DeFries R, Asner GP, Barford C, Bonan G, Carpenter SR, Chapin FS, Coe MT, Daily GC, Gibbs HK (2005). Global consequences of land use. Science.

[2] Alexander P, Brown C, Arneth A, Dias C, Finnigan J, Moran D. Rounsevell MDA: Could consumption of insects, cultured meat or imitation meat reduce global agricultural land use? Global Food Security 2017:22–32.

[3] Muller A, Ferré M, Engel S, Gattinger A, Holzkämper A, Huber R, Müller M, Six J (2017). Can soil-less crop production be a sustainable option for soil conservation and future agriculture?. Land Use Pol.

[4] MacFalane A, Martin G (2002). The glass bathyscaphe: how glass changed the world.

[5] Shamshiri RR (2021). Next-generation greenhouses for food security.

[6] Poore J, Nemecek T (2018). Reducing food’s environmental impacts through producers and consumers. Science.

[7] Despommier DD (2010). The vertical farm : feeding the world in the 21st century.

[8] Kozai T, Niu G, Takagaki M, editors. Plant Factory; An Indoor Vertical Farming System for Efficient Quality Food Production (2nd. Edition). Amsterdam: Academic Press; 2019.

[9] van Delden SH, SharathKumar M, Butturini M, Graamans LJA, Heuvelink E, Kacira M, Kaiser E, Klamer RS, Klerkx L, Kootstra G (2021). Current status and future challenges in implementing and upscaling vertical farming systems. Nat Food.

[10] Monje O, Bugbee B (1998). Adaptation to high CO_2_ concentration in an optimal environment: radiation capture, canopy quantum yield and carbon use efficiency. Plant Cell Environ.

[11] Asseng S, Guarin JR, Raman M, Monje O, Kiss G, Despommier DD, Meggers FM, Gauthier PPG (2020). Wheat yield potential in controlled-environment vertical farms. Proc Natl Acad Sci U S A.

[12] Van Gerrewey T, Boon N (2022). Geelen D: Vertical farming: the only way is up?. Agronomy.

[13] Pratty F. Energy costs create headwinds for vertical farms (Financial Times; May 4 2023). 2023. Available from: https://www.ft.com/content/bfb334fe-d53e-405c-956a-304d5abcd9ee.

[14] Dorr E, Goldstein B, Horvath A, Aubry C, Gabrielle B (2021). Environmental impacts and resource use of urban agriculture: a systematic review and meta-analysis. Environ Res Lett.

[15] Bunge AC, Wood A, Halloran A, Gordon LJ (2022). A systematic scoping review of the sustainability of vertical farming, plant-based alternatives, food delivery services and blockchain in food systems. Nat Food.

[16] Kobayashi Y, Kotilainen T, Carmona-Garcia G, Leip A, Tuomisto HL (2022). Vertical farming: a trade-off between land area need for crops and for renewable energy production. J Clean Prod.

[17] Stranges AN: A history of the Fischer-Tropsch synthesis in Germany 1926–45. In: *229th National Meeting of the American-Chemical-Society: Mar 13–17 2005; San Diego, CA*. 2006: 1–27.

[18] Cai T, Sun HB, Qiao J, Zhu LL, Zhang F, Zhang J, Tang ZJ, Wei XL, Yang JG, Yuan QQ (2021). Cell-free chemoenzymatic starch synthesis from carbon dioxide. Science.

[19] Davis SJ, Alexander K, Moreno-Cruz J, Hong CP, Shaner M, Caldeira K, McKay I (2024). Food without agriculture Nat Sustain.

[20] Palyi G (2019). Biological chirality.

[21] Ritchie H, Roser M. Land Use. 2019. Available from: https://ourworldindata.org/land-use.

[22] Marcellin E, Angenent LT, Nielsen LK, Molitor B (2022). Recycling carbon for sustainable protein production using gas fermentation. Curr Opin Biotech.

[23] Sillman J, Nygren L, Kahiluoto H, Ruuskanen V, Tamminen A, Bajamundi C, Nappa M, Wuokko M, Lindh T, Vainikka P (2019). Bacterial protein for food and feed generated via renewable energy and direct air capture of CO_2_: Can it reduce land and water use?. Glob Food Secur-Agr.

[24] Köpke M, Simpson SD (2020). Pollution to products: recycling of ‘above ground’ carbon by gas fermentation. Curr Opin Biotech.

[25] Liu C, Colón BC, Ziesack M, Silver PA, Nocera DG (2016). Water splitting-biosynthetic system with CO_2_ reduction efficiencies exceeding photosynthesis. Science.

[26] Ritchie H, Rosado P, Roser M. Meat and dairy production. 2023. Available from: https://ourworldindata.org/meat-production.

[27] Godfray HCJ, Aveyard P, Garnett T, Hall JW, Key TJ, Lorimer J, Pierrehumbert RT, Scarborough P, Springmann M, Jebb SA (2018). Meat consumption, health, and the environment. Science.

[28] Steinfeld H, Gerber P, Wassenaar TD, Castel V, Rosales M, Rosales M, et al. Livestock's Long Shadow. Rome: FAO; 2006.

[29] Xu XM, Sharma P, Shu SJ, Lin TS, Ciais P, Tubiello FN, Smith P, Campbell N, Jain AK (2021). Global greenhouse gas emissions from animal-based foods are twice those of plant-based foods. Nat Food.

[30] He J, Evans NM, Liu HZ, Shao SQ (2020). A review of research on plant-based meat alternatives: driving forces, history, manufacturing, and consumer attitudes. Comprehensive Reviews in Food Science and Food Safety.

[31] Good Food Institute. State of Global Policy. Public Investment in Alternative Proteins to Feed a Growing World. New York: GFI; 2023.

[32] Impossible Foods. 2023. Available from: https://impossiblefoods.com/food/.

[33] Beyond Meat. 2023. Available from: https://www.beyondmeat.com/en-GB/.

[34] McClements DJ, Grossmann L. Next-generation plant-based foods: challenges and opportunities. Annual Review of Food Science and Technology 2023;15:10.1146/annurev-food-072023-034414. 10.1146/annurev-food-072023-034414 37963430

[35] McClements DJ, Grossmann L (2022). Next-generation plant-based foods: design, production, and properties.

[36] Mariotti F (2017). Vegetarian and plant-based diets in health and disease prevention.

[37] Dimina L, Remond D, Huneau JF, Mariotti F (2022). Combining plant proteins to achieve amino acid profiles adapted to various nutritional objectives - an exploratory analysis using linear programming. Front Nutr.

[38] Akharume FU, Aluko RE, Adedeji AA (2021). Modification of plant proteins for improved functionality: a review. Comprehensive Reviews in Food Science and Food Safety.

[39] Aschemann-Witzel J, Gantriis RF, Fraga P, Perez-Cueto FJA (2021). Plant-based food and protein trend from a business perspective: markets, consumers, and the challenges and opportunities in the future. Crit Rev Food Sci.

[40] Fraser EDG, Rimas A (2010). Empires of food.

[41] Linder T (2023). Beyond agriculture - how microorganisms can revolutionize global food production. ACS Food Science & Technology.

[42] Graham AE, Ledesma-Amaro R (2023). The microbial food revolution Nat Commun.

[43] Banks M, Johnson R, Giver L, Bryant G, Guo M (2022). Industrial production of microbial protein products. Curr Opin Biotech.

[44] Yap WS, Choudhury D, Suntornnond R (2023). Towards biomanufacturing of cultured meat. Trends Biotechnol.

[45] Wiebe MG (2002). Myco-protein from *Fusarium venenatum*: a well-established product for human consumption. Appl Microbiol Biot.

[46] Ritala A, Häkkinen ST, Toivari M, Wiebe MG (2009). Single cell protein-state-of-the-art, industrial landscape and patents 2001–2016. Front Microbiol.

[47] Yuan SF, Alper HS (2019). Metabolic engineering of microbial cell factories for production of nutraceuticals. Microb Cell Fact.

[48] Lv XQ, Wu YK, Gong MY, Deng JY, Gu Y, Liu YF, Li JH, Du GC, Ledesma-Amaro R, Liu L (2021). Synthetic biology for future food: research progress and future directions. Future Foods.

[49] Wang YY, Liu LX, Jin ZX, Zhang DW (2021). Microbial cell factories for green production of vitamins. Frontiers in Bioengineering and Biotechnology.

[50] Kazer J, Orfanos G, Gallop C (2022). Quorn footprint comparison report.

[51] Watson E. ENOUGH raises $43.6m to scale up mycoprotein production. 2023. Available from: https://agfundernews.com/alt-protein-isnt-dead-yet-says-enough-as-it-raises-43-6m-to-scale-up-mycoprotein-production.

[52] Javourez U, O’Donhue M, Hamelin L (2021). Waste-to-nutrition: a review of current and emerging conversion pathways. Biotechnol Adv.

[53] Woern C, Grossmann L (2023). Microbial gas fermentation technology for sustainable food protein production. Biotechnol Adv.

[54] Gregorio NE, Levine MZ, Oza JP (2019). A user’s guide to cell-free protein synthesis. Methods and Protocols.

[55] Churchill WLS. Fifty years hence. The Strand Magazine.1931: December.

[56] Terazono E. Record sums flow into alternative meats in first quarter. 2020. Available from: https://www.ft.com/content/edf2db2f-bbcc-42f2-8aa4-233c0c46b4c6.

[57] United Nations Environment Programme. What’s Cooking? An assessment of the potential impacts of selected novel alternatives to conventional animal products. Nairobi: UNEP; 2023.

[58] Yu D. “Eat Just” to scale up cultured meat production on gaining new regulatory approval in Singapore. 2023. Available from: https://www.forbes.com/sites/douglasyu/2023/01/18/eat-just-to-scale-up-cultured-meat-production-on-gaining-new-regulatory-approval-in-singapore/.

[59] Hong TK, Shin DM, Choi J, Do JT, Han SG (2021). Current issues and technical advances in cultured meat production: a review. Food Science of Animal Resources.

[60] Post MJ, Levenberg S, Kaplan DL, Genovese N, Fu JA, Bryant CJ, Negowetti N, Verzijden K, Moutsatsou P (2020). Scientific, sustainability and regulatory challenges of cultured meat. Nat Food.

[61] Bhat ZF, Morton JD, Mason SL, Bekhit AEA, Bhat HF (2019). Technological, regulatory, and ethical aspects of i*n vitro* meat: a future slaughter-free harvest. Comprehensive Reviews in Food Science and Food Safety.

[62] Stephens N, Di Silvio L, Dunsford I, Ellis M, Glencross A, Sexton A (2018). Bringing cultured meat to market: technical, socio-political, and regulatory challenges in cellular agriculture. Trends Food Sci Tech.

[63] Wood P, Thorrez L, Hocquette JF, Troy D, Gagaoua M (2023). Cellular agriculture”: current gaps between facts and claims regarding “cell-based meat. Anim Front.

[64] Fraser EDG, Kaplan DL, Newman L, Yada RY (2023). Cellular agriculture.

[65] Lee DY, Lee SY, Yun SH, Jeong JW, Kim JH, Kim HW, Choi JS, Kim GD, Joo ST, Choi I (2022). Review of the current research on fetal bovine serum and the development of cultured meat. Food Science of Animal Resources.

[66] Thorrez L, Vandenburgh H (2019). Challenges in the quest for ‘clean meat’. Nat Biotechnol.

[67] FAOSTAT. Food and agriculture commodities production. 2024. Available from: http://faostat.fao.org/default.aspx.

[68] Reynolds M: Insiders reveal major problems at lab-grown-meat startup Upside Foods [https://www.wired.com/story/upside-foods-lab-grown-chicken/]. Accessed 24 January 2024.

[69] Ben-Arye T, Shandalov Y, Ben-Shaul S, Landau S, Zagury Y, Ianovici I, Lavon N, Levenberg S (2020). Textured soy protein scaffolds enable the generation of three-dimensional bovine skeletal muscle tissue for cell-based meat. Nat Food.

[70] Tuomisto HL, de Mattos MJT (2011). Environmental impacts of cultured meat production. Environ Sci Technol.

[71] Risner R, Kim Y, Nguyen C, Siegel JB, Spang ES. Environmental impacts of cultured meat: a cradle-to-gate life cycle assessment. BioRxiv 2023:537778. 10.1021/acsfoodscitech.4c00281 PMC11744764 39840401

[72] Cornet SHV, Snel SJE, Schreuders FKG, van der Sman RGM, Beyrer M, van der Goot AJ (2022). Thermo-mechanical processing of plant proteins using shear cell and high-moisture extrusion cooking. Crit Rev Food Sci.

[73] Grossmann L, Weiss J: Alternative protein sources as technofunctional food ngredients. In: Annual Review of Food Science and Technology. Edited by Doyle M, McClements DJ; 2021: 93–117. 10.1146/annurev-food-062520-093642 33472014

[74] Wen Y, Che QT, Kim HW, Park HJ (2021). Potato starch altered the rheological, printing, and melting properties of 3D-printable fat analogs based on inulin emulsion-filled gels. Carbohyd Polym.

[75] Chen YY, Zhang M, Bhandari B (2011). 3D printing of steak-like foods based on textured soybean protein. Foods.

[76] Arora S, Kataria P, Nautiyal M, Tuteja I, Sharma V, Ahmad F, Haque S, Shahwan M, Capanoglu E, Vashishth R (2023). Comprehensive review on the role of plant protein as a possible meat analogue: framing the future of meat. ACS Omega.

[77] Sharma H. A detailed chemistry of coffee and its analysis. In: Castanheira DT, editor. Coffee Production and Research. London: Intechopen; 2020.

[78] Ridder M: Coffee consumption worldwide from 2012/13 to 2021/22 with a forecast to 2022/23 [https://www.statista.com/statistics/292595/global-coffee-consumption/]. Accessed 24 January 2024.

[79] Prendergast M. Uncommon Grounds: The History of Coffee and How it Transformed Our World. New York: Basic Books; 2010.

[80] Mridul A: Beanless coffee: 7 businesses making molecular, cell-based coffee a reality [https://www.greenqueen.com.hk/beanless-coffee-startups-lab-grown-molecular-cell-based/]. Accessed 24 January 2024.

[81] Aisala H, Karkkainen E, Jokinen I, Seppanen-Laakso T, Rischer H (2023). Proof of concept for cell culture-based coffee. J Agric Food Chem.

[82] Mehta BM. Chemical composition of milk and milk products. In: Handbook of Food Chemistry*.* Edited by Cheung PCK, Mehta BM. Berlin, Heidelberg: Springer Berlin Heidelberg; 2015: 511–553.

[83] Vanga SK, Raghavan V (2018). How well do plant based alternatives fare nutritionally compared to cow’s milk?. Journal of Food Science and Technology-Mysore.

[84] Sethi S, Tyagi SK, Anurag RK (2016). Plant-based milk alternatives an emerging segment of functional beverages: a review. Journal of Food Science and Technology-Mysore.

[85] McClements DJ, Newman E, McClements IF (2019). Plant-based milks: a review of the science underpinning their design, fabrication, and performance. Comprehensive Reviews in Food Science and Food Safety.

[86] Järviö N, Parviainen T, Maljanen NL, Kobayashi Y, Kujanpää L, Ercili-Cura D, Landowski CP, Ryynänen T, Nordlund E, Tuomisto HL (2021). Ovalbumin production using *Trichoderma reesei* culture and low-carbon energy could mitigate the environmental impacts of chicken-egg-derived ovalbumin. Nat Food.

[87] Aro N, Ercili-Cura D, Andberg M, Silventoinen P, Lille M, Hosia W, Nordlund E, Landowski CP (2023). Production of bovine beta-lactoglobulin and hen egg ovalbumin by *Trichoderma reesei* using precision fermentation technology and testing of their techno-functional properties. Food Res Int.

[88] Boukid F, Gagaoua M (2022). Vegan egg: a future-proof food ingredient?. Foods.

[89] United Nations (2022). World Population Prospects 2022.

[90] Lutz W (2021). Advanced introduction to demography.

[91] Tilman D, Fargione J, Wolff B, D’Antonio C, Dobson A, Howarth R, Schindler D, Schlesinger WH, Simberloff D, Swackhamer D (2001). Forecasting agriculturally driven global environmental change. Science.

[92] van Dijk M, Morley T, Rau ML, Saghai Y (2021). A meta-analysis of projected global food demand and population at risk of hunger for the period 2010–2050. Nat Food.

[93] de Ribou SD, Douam F, Hamant O, Frohlich MW, Negrutiu J (2013). Plant science and agricultural productivity: why are we hitting the yield ceiling?. Plant Sci.

[94] Godfray HCJ, Beddington JR, Crute IR, Haddad L, Lawrence D, Muir JF, Pretty J, Robinson S, Thomas SM, Toulmin C (2010). Food security: the challenge of feeding 9 billion people. Science.

[95] Conway G (1997). The doubly green revolution.

[96] Gustavsson J, Cederberg C, Sonesson U, van Otterdijk R, Meybeck A (2011). Global food losses and food waste.

[97] Mbow C, Rosenzweig C, Barioni LG, Benton TC, Herrero M, Krishnapillai M, Liwenga E, Pradhan P, Rivera-Ferre MG, Sapkota T et al. Food security. In: Climate Change and Land: an IPCC special report on climate change, desertification, land degradation, sustainable land management, food security, and greenhouse gas fluxes interrestrial ecosystems*.* Edited by Shukla PA; 2019.

[98] Alexander P, Brown C, Arneth A, Finnigan J, Rounsevell MDA (2016). Human appropriation of land for food: the role of diet. Global Environ Chang.

[99] Rockefeller Foundation: True cost of food: measuring what matters to transform the U.S. food system. New York: Rockefeller Foundation; 2021.

[100] Damania R, Balseca E, de Fontaubert C, Gill J, Kim K, Rentschler J, Russ J, Zaveri E (2023). Detox development: repurposing environmentally harmful subsidies.

[101] Vallone S, Lambin EF (2023). Public policies and vested interests preserve the animal farming status quo at the expense of animal product analogs. One Earth.

[102] Mac R. Unilever drops mayo lawsuit against egg-replacing startup Hampton Creek [https://www.forbes.com/sites/ryanmac/2014/12/18/unilever-drops-mayo-lawsuit-against-egg-replacing-startup-hampton-creek/]. Accessed 24 January 2024.

[103] Bryant CJ (2020). Culture, meat, and cultured meat. J Anim Sci.

[104] Bryant RL, Goodman MK (2004). Consuming narratives: the political ecology of ‘alternative’ consumption. T I Brit Geogr.

[105] Caldwell JM (2021). Are we ready for cultured meat?. Food Technol.

[106] Laestadius LI, Caldwell MA (2015). Is the future of meat palatable? Perceptions of *in vitro* meat as evidenced by online news comments. Public Health Nutr.

[107] Mancini MC, Antonioli F (2020). To what extent are consumers’ perception and acceptance of alternative meat production systems affected by information?. The case of cultured meat Animals.

[108] Siegrist M, Hartmann C (2020). Consumer acceptance of novel food technologies. Nat Food.

[109] Sexton AE, Garnett T, Lorimer J (2019). Framing the future of food: the contested promises of alternative proteins. Environment and Planning E-Nature and Space.

